# Effects of copper and copper oxide nanoparticles on cyanobacterium *Nostoc linckia*: an experimental study

**DOI:** 10.3389/fmicb.2025.1553857

**Published:** 2025-02-24

**Authors:** Liliana Cepoi, Ludmila Rudi, Tatiana Chiriac, Ana Valuta, Svetlana Codreanu, Tatiana Mitina, Liviu Codreanu

**Affiliations:** ^1^Ficobiotechnology Laboratory, Institute of Microbiology and Biotechnology, Technical University of Moldova, Chisinau, Moldova; ^2^Water Chemistry Laboratory, Institute of Chemistry, State University of Moldova, Chisinau, Moldova

**Keywords:** copper nanoparticles, copper oxide nanoparticles, *Nostoc linckia*, biomass amount, biochemical changes, copper accumulation

## Abstract

**Introduction:**

Copper nanoparticles (CuNPs) and copper oxide nanoparticles (CuONPs) are increasingly explored for their biological interactions with various organisms, including cyanobacteria, due to their unique properties and potential applications. This study investigates the effects of CuNPs and CuONPs on the cyanobacterium *Nostoc linckia* (Roth) Born et Flah CNMN-CB-03, focusing on biomass accumulation, biochemical content, pigment composition, and microscopic structural changes.

**Methods:**

*Nostoc linckia* cultures were exposed to CuNPs and CuONPs at concentrations ranging from 0.1 to 30 mg/L. The impact on biomass, protein, pigment, lipid content, malondialdehyde (MDA) levels, and bioaccumulation of copper was assessed, alongside microscopic analysis to observe any structural modifications in trichomes.

**Results:**

The effects of CuNPs and CuONPs on *Nostoc linckia* were distinct. Under high concentrations of CuNPs exposure, reductions in biomass, protein content, and pigments were observed, whereas lipid and MDA content increased significantly. Similarly, CuONPs caused a marked increase in lipid and MDA levels, suggesting oxidative stress despite the comparatively moderate alterations in other biochemical parameters. Both nanoparticle types, however, caused notable bioaccumulation of copper and structural modification in *Nostoc linckia* cells expressed in trichome fragmentation, chromaticity changes, and variations in heterocyst numbers and size in treated samples.

**Conclusion:**

CuNPs and CuONPs exhibit differential effects on *Nostoc linckia*, influencing biochemical composition, pigment profiles, and cellular structure. These findings contribute to understanding nanoparticle interactions with cyanobacteria and highlight the distinct impact of nanoparticle composition on microbial systems.

## 1 Introduction

The rapid advancement of nanotechnology has driven the widespread application of metal-based nanoparticles, including copper (CuNPs) and copper oxide nanoparticles (CuONPs), owing to their unique physicochemical properties and versatility across various fields. CuNP-based fertilizers and herbicides show potential in agriculture, as their nanoscale size facilitates efficient plant absorption. Furthermore, CuNPs are promising in food packaging, where they inhibit spoilage microorganisms, with their integration into agar-based materials significantly extending food shelf life (Rai et al., 2018). Similarly, CuONPs are extensively utilized in agriculture, medicine, and environmental management due to their antimicrobial properties, high reactivity, and stability, enhancing plant growth, extending food preservation, and enabling applications in water purification, catalysis, and energy storage (Bonilla-Bird et al., 2020; Ahamed et al., 2014).

Despite these benefits, CuNPs and CuONPs exhibit dual effects on biological systems. At high concentrations, they induce oxidative stress, disrupt cellular processes, and inhibit microorganism growth. Conversely, low concentrations can stimulate growth, enhance pigment production, and improve metabolic activity. This duality highlights their potential in promoting beneficial microorganisms, such as microalgae and cyanobacteria, while controlling harmful ones, but also underscores their ecological risks (Agathokleous et al., 2019; Tortella et al., 2024). Thus, a thorough evaluation of their environmental and biological impacts is critical for safe and effective utilization (Arif et al., 2018).

The interaction of CuNPs and CuONPs with microorganisms, especially cyanobacteria and microalgae, is an area of growing interest due to their ecological importance and biotechnological applications (Wan et al., 2018; Huang et al., 2022; Barreto et al., 2021; Lau et al., 2022; Solomonova E. S. et al., 2023; Solomonova E. et al., 2023). A key challenge lies in understanding their movement, transformation, and bioavailability in dynamic environments. Research often assesses nanoparticle toxicity based on their direct interaction with microalgae and cyanobacteria, considering factors such as surface characteristics, metal ion release, and hazardous by-products generated during NP-microorganism interactions (Déniel et al., 2019; Wan et al., 2018). However, long-term effects of low-concentration NP exposure remain poorly understood, highlighting the need for comprehensive studies on NP distribution, dispersion, and adsorption mechanisms (Nguyen et al., 2020).

As primary producers in aquatic ecosystems, microalgae and cyanobacteria exhibit complex responses to NP exposure, primarily driven by oxidative stress. Adaptive mechanisms employed by these organisms to mitigate NP-induced stress open possibilities for leveraging such responses to promote beneficial microalgae or cyanobacteria growth while controlling harmful blooms (Agathokleous et al., 2019; Solomonova E. S. et al., 2023; Solomonova E. et al., 2023; Vargas-Estrada et al., 2020; Sibi et al., 2017; Mahfooz et al., 2019; Kumar et al., 2023).

The cyanobacterium *Nostoc linckia* plays a vital ecological and biotechnological role by fixing atmospheric nitrogen, enhancing soil fertility, and producing valuable bioactive compounds such as polysaccharides and pigments with applications in agriculture, medicine, and the food industry (Touloupakis et al., 2022; El-fayoumy et al., 2023; Ramadan et al., 2021). Moreover, it serves as a model organism for studying stress responses and bioaccumulation, making it an important resource for environmental and biotechnological research. Understanding how CuNPs and CuONPs affect *Nostoc linckia* is crucial for evaluating their environmental impact and harnessing their potential in practical applications.

Thus, this study aims to determine the effects of CuNPs and CuONPs, applied at concentrations ranging from 0.1 to 30 mg/L, on the cyanobacterium *Nostoc linckia*, strain CNMN-CB-03.

## 2 Materials and methods

### 2.1 Copper and copper oxide nanoparticles

The two types of nanoparticles used in this research were procured from Sigma-Aldrich (Merck KGaA, Darmstadt, Germany). The size of the copper nanoparticles (CuNPs) was determined using transmission electron microscopy (TEM) and averaged 25 nm. The CuNPs had a purity of 99.5%, and their product code is 774081. The average size of the copper oxide nanoparticles (CuONPs), also determined via TEM, was 50 nm, with a product code of 544868. Nanoparticle in deionized water were subjected to ultrasonic treatment at a frequency of 22 kHz, an intensity of 7 W/cm^2^, and a duration of 20 min. The ultrasonic treatment aimed to disperse the nanoparticles and prevent the formation of aggregates.

### 2.2 *Nostoc linckia* strain, nutrient medium and cultivation conditions

The cyanobacterial strain *Nostoc linckia* (Roth) Born et Flah CNMN-CB-03 was used as the object of study. This strain is preserved in the National Collection of Non-Pathogenic Microorganisms at the Institute of Microbiology, Technical University of Moldova. The mineral nutrient medium used for its cultivation had the following composition: macronutrients (g/L)—KNO_3_: 0.5, K_2_HPO_4_: 0.45, NaHCO_3_: 0.05, MgSO_4_·7H_2_O: 0.1, CaCl_2_: 0.11; and micronutrients (mg/L)—ZnSO_4_·7H_2_O: 0.05, MnSO_4_: 2.0, H_3_BO_3_: 0.85, (NH_4_)_6_Mo_7_O_24_·4H_2_O: 2.25, FeSO_4_·7H_2_O: 4.0, Co(NO_3_)_2_·H_2_O: 0.009, EDTA: 4.75. Cyanobacteria were cultivated in 100 mL Erlenmeyer flasks with a culture volume of 50 mL under the following conditions: medium pH 6.8–7.2, temperature 25–27°C, continuous illumination at a light intensity of 55 μmol photons/m^2^/s, and slow periodic stirring (Cepoi et al., 2022). Copper and copper oxide nanoparticles were added to the nutrient medium on the first day of cultivation, in 5 concentrations: 0.1; 1.0; 10.0; 20.0; 30.0 mg/L. The growth cycle lasted 12 days.

### 2.3 Determination of the amount of biomass

The biomass quantity of *Nostoc linckia* in both experimental and control variants was determined based on a calibration curve expressing the relationship between the culture absorbance at 590 nm and the dry cell mass fraction in the analyzed suspension. This parameter is expressed in g/L. The linearity range of the calibration curve is between 0.1 and 0.4 absorbance units.

### 2.4 Sample preparation for analysis

The biomass of *Nostoc linckia* is separated by centrifugation at 5,000 g for 10 min. The biomass is washed with distilled water and centrifuged again. It is then standardized in distilled water to obtain a suspension with a concentration of 10 mg/mL. The culture liquid and the water used for washing are collected for copper determination. The biomass samples undergo repeated freeze–thaw cycles (6 cycles), with freezing performed at −20°C and thawing at room temperature. The standardized samples are stored at −20°C.

### 2.5 Determination of the amount of copper

The metal content was determined using flame atomic absorption spectrophotometry. Copper content was determined at a wavelength of 324.7 nm in an acetylene-air flame. The calibration graph was constructed from the certified reference material Copper standard solution manufactured by Sigma Aldrich. The linearity range of the graph was from 0.01 to 4 mg/L. The correlation coefficient of the calibration graph was 0.998 (Duca et al., 2019).

### 2.6 Protein quantification in biomass

The protein content in the biomass was determined using the Lowry method (Lowry et al., 1951). Protein extraction was performed by treating the biomass with a 0.1 N NaOH solution for 30 min. For 10 mg of biomass, 0.9 mL of 0.1 N NaOH solution was used. After the extraction process, 0.2 mL of the hydrolysate was mixed with 0.8 mL of distilled water, followed by the addition of 1.5 mL of complex reagent. This complex reagent consisted of 49 mL of 2% Na₂CO₃ solution in 0.1 N NaOH and 1 mL of 0.5% CuSO₄ solution in 1% sodium potassium tartrate (C₄H₄Na₂O₆). The mixture was left at room temperature for 10 min, after which 0.5 mL of Folin–Ciocalteu reagent, diluted at a 1:3 ratio, was added. The reaction mixture was allowed to stand for 40 min, and the absorbance of the solution was measured at a wavelength of 750 nm. The protein content was calculated using a calibration curve constructed with bovine serum albumin as the reference standard.

### 2.7 Determination of phycobiliprotein content

The phycobiliprotein content in the biomass of *Nostoc linckia* was determined spectrophotometrically using a modified version of the method described by Bennett and Bogorad (1973). For this purpose, 1.0 mL of standardized biomass with a concentration of 10 mg/mL, previously subjected to freeze–thaw cycles, was centrifuged at 11,000 g for 20 min at 4°C. The absorbance of the samples was recorded at wavelengths of 565 nm, 620 nm, and 650 nm, corresponding to the specific absorption maxima of phycoerythrin (PE), phycocyanin (PC), and allophycocyanin (APC), respectively. The quantitative content of the phycobiliproteins was calculated using the equations proposed by Bennett and Bogorad (1973).

### 2.8 Determination of carbohydrate content

The carbohydrate content in the biomass of *Nostoc linckia* was determined using a spectrophotometric method based on the specific reaction with Anthrone reagent in an acidic medium. To 20 μL of standardized *Nostoc* biomass suspension, 2 mL of 0.5% Anthrone reagent in 66% sulfuric acid was added. Hydrolysis was performed for 10 min in a boiling water bath. After hydrolysis, the mixture was cooled and incubated for 30 min at room temperature. The absorbance of the samples was measured at a wavelength of 620 nm (Santiago-Díaz et al., 2021). The carbohydrate content was calculated based on a calibration curve constructed using glucose as the reference standard. The detection range was 0.02–0.10 μg/mL.

### 2.9 Determination of chlorophyll α and carotenoid content

The chlorophyll a and total carotenoid content were determined spectrophotometrically after pigment extraction in an ethanolic solvent. For this purpose, 10 mg of *Nostoc linckia* biomass was suspended in 1 mL of 96% ethanol and continuously stirred for 12 h at room temperature. The samples were then centrifuged at 5,000 g for 10 min. The absorbance of the supernatant was measured at wavelengths of 450 nm, 649 nm, and 665 nm. The pigments content was calculated using the equations proposed by Lichtenthaler and Wellburn (Lichtenthaler and Wellburn, 1983). The pigments content is expressed as a percentage relative to the dry biomass.

### 2.10 Determination of lipid content

The lipid content in the biomass of *Nostoc linckia* was determined spectrophotometrically using the phosphovanillin reagent (Park et al., 2016). The reagent was prepared by dissolving 0.75 g of vanillin in 125 mL of distilled water, followed by the addition of 500 mL of 85% phosphoric acid. For the analysis, 10 mg of biomass was treated with 1.0 mL of a chloroform-ethanol mixture in a 9:1 (v/v) ratio to extract the lipids. The lipid extract was separated from the residual biomass and dried. The dry residue was treated with 1 mL of sulfuric acid and heated for 10 min at high temperature. After cooling, 0.1 mL of the hydrolysate was reacted with 2.9 mL of phosphovanillin reagent, prepared by dissolving 1.2 mg of vanillin in 1.0 mL of 68% phosphoric acid. The absorbance was measured at 520 nm after 30 min of incubation. The lipid content was calculated based on a calibration curve constructed using pure oleic acid.

### 2.11 Determination of malondialdehyde content

The malondialdehyde (MDA) content was determined spectrophotometrically using the thiobarbituric acid (TBA) assay. This method is based on the formation of MDA-TBA reactive products. To 1 mL of standardized biomass suspension at a concentration of 10 mg/mL, 3 mL of 0.67% TBA solution in 20% trichloroacetic acid (TCA) was added. The mixture was incubated at 100°C for 20 min, then cooled and centrifuged at 3,000 g for 15 min. The absorbance of the supernatant was measured at 535 nm, which corresponds to the maximum absorption of the MDA-TBA complex, and at 600 nm to correct for nonspecific pigmentation. The MDA content, expressed as ng/g of biomass, was calculated using the molar extinction coefficient of the MDA-TBA complex (*ε* = 1.56 × 10^5^ M^−1^ cm^−1^) (Rudi et al., 2025).

### 2.12 Statistical analysis

The experiments were conducted in three independent replicates. The experimental results were expressed as mean value ± standard deviation (SD). To evaluate differences between the control and experimental conditions, Student’s *t*-test was applied. A statistical significance level of *p* ≤ 0.05 was considered relevant.

## 3 Results

### 3.1 The effect of copper and copper oxide nanoparticles on biomass accumulation in *Nostoc linckia* culture

**Figure 1:**
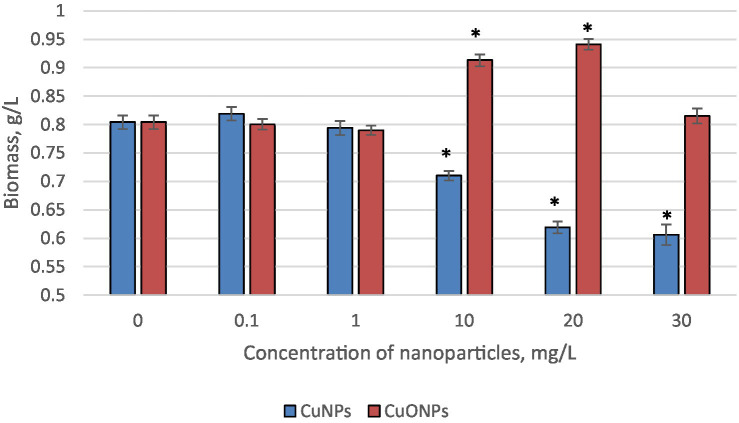
The effect of CuNPs and CuONPs on the biomass quantity of *Nostoc linckia* (in *g* of dry substance per liter). *p < 0.05.

The results concerning the biomass accumulation of *Nostoc linckia* at the end of the growth cycle, upon the addition of two types of nanoparticles—copper (CuNPs) and copper oxide (CuONPs)—each at concentrations of 0.1, 1.0, 10.0, 20.0, and 30.0 mg/L, are presented in Figure 1.

At concentrations of 0.1 and 1.0 mg/L, neither type of nanoparticle caused any changes in the amount of biomass accumulated by the culture at the end of the growth cycle. Starting at a concentration of 10.0 mg/L, the effects of CuNPs and CuONPs began to differ. Specifically, copper nanoparticles at 10.0 mg/L caused a statistically significant reduction in biomass by 11.7% compared to the control group (*p* = 0.049), while CuONPs at the same concentration induced a 13.6% increase in biomass compared to the control (*p* = 0.0005). A similar trend was observed at a concentration of 20.0 mg/L: CuNPs caused a 23.0% reduction in biomass relative to the control (*p* = 0.019), whereas CuONPs led to a 17.0% increase in biomass (*p* = 0.03). At 30.0 mg/L, copper nanoparticles strongly inhibited biomass accumulation, with a reduction of 24.6% compared to the control (*p* = 0.0006). In contrast, the stimulating effect of CuONPs disappeared at this concentration, and the biomass level was comparable to that of the control.

### 3.2 Copper accumulation in biomass of *Nostoc linckia* cultivated with CuNPs and CuONPs

The results regarding copper accumulation in the biomass of *Nostoc linckia* during cultivation in the presence of copper and copper oxide nanoparticles are presented in Table 1.

**Table 1:** Copper accumulation in the biomass of *Nostoc linckia* depending on the concentration of CuNPs and CuONPs.

Type of NPs	NPs concentration, mg/L	Cu content, mg/L	Cu remaining in the culture liquid, mg/L	% of Cu accumulation
CuNPs	0.1	0.1	BDL*	100
	1.0	1.0	BDL*	100
	10.0	10.0	BDL*	100
	20.0	20.0	0.470 ± 0.026	97.063
	30.0	30.0	0.513 ± 0.047	97.875
CuONPs	0.1	0.08	BDL*	100
	1.0	0.80	0.095 ± 0.013	90.500
	10.0	8.00	0.353 ± 0.040	96.467
	20.0	15.97	1.080 ± 0.075	94.600
	30.0	23.96	1.233 ± 0.080	95.889

*BDL: Below detection limit of the method (<0.01 mg/L).

Copper accumulation was highly efficient for both types of nanoparticles, as indicated by the residual copper concentrations in the culture liquid at the end of the growth cycle, which showed a metal removal rate of 90.5 to 100.0%. Copper nanoparticles at concentrations of 0.1, 1.0, and 10.0 mg/L, as well as copper oxide nanoparticles at 0.1 mg/L, were completely taken up by the Nostoc biomass. At higher concentrations, copper removal from the culture liquid ranged between 90.5 and 97.9% of the initial amount introduced into the medium.

### 3.3 Modification of pigment content in *Nostoc linckia* biomass under the influence of Cu and CuO nanoparticles

**Figure 2:**
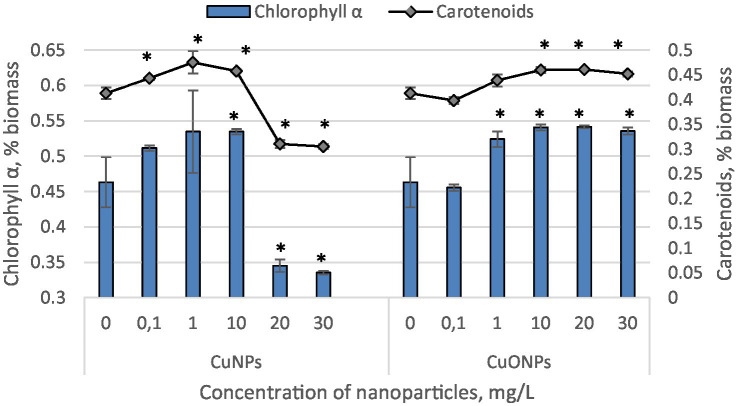
The effect of CuNPs and CuONPs on the quantity of chlorophyll α and carotenoids in *Nostoc linckia* biomass (in % of dry biomass). *p < 0.05.

The results obtained from quantifying the content of chlorophyll α and carotenoids in *Nostoc linckia* biomass during cultivation in the presence of different concentrations of copper and copper oxide nanoparticles are presented in Figure 2. As seen, copper nanoparticles induced a statistically significant increase in carotenoid content at concentrations of 0.1, 1.0, and 10.0 mg/L (up to 15.1% at 1.0 mg/L CuNPs, *p* = 0.0186), but a pronounced decrease at concentrations of 20.0 and 30.0 mg/L, with reductions of 24.8 and 26.1%, respectively, compared to the control. A similar pattern of change was observed for chlorophyll α, except that a significant increase in pigment content was only observed at a concentration of 10.0 mg/L CuNPs. The differences compared to the control for concentrations of 0.1 and 1.0 mg/L were not statistically significant. In contrast, copper oxide nanoparticles induced a significant increase in the content of both pigments at concentrations of 10.0, 20.0, and 30.0 mg/L. The highest values were obtained at a concentration of 20.0 mg/L, with chlorophyll content increasing by 16.9% and carotenoids by 11.6% compared to the control.

**Figure 3:**
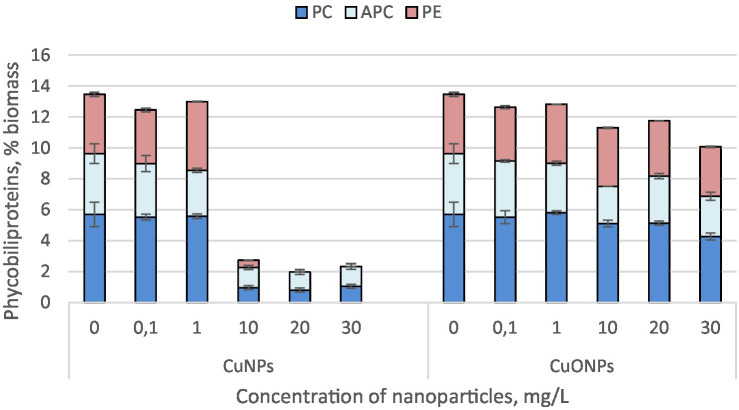
The effect of CuNPs and CuONPs on the quantity of phycobiliproteins in *Nostoc linckia* biomass (in % of dry biomass). PC, phycocyanin; APC, allophycocyanin; PE, phycoerythrin.

The quantity of phycobiliproteins in the biomass of *Nostoc linckia* from both experimental and control variants is presented in Figure 3. The *Nostoc* biomass contains three phycobilin pigments: phycocyanin, allophycocyanin, and phycoerythrin.

The most significant changes were observed at copper nanoparticle concentrations of 10.0, 20.0, and 30.0 mg/L. At these concentrations, the phycobiliprotein content in cyanobacterial biomass decreased by more than sixfold compared to the control, constituting only 1.96–2.71% of the biomass, whereas in the control, phycobiliproteins accounted for 13.45%. Additionally, at CuNP concentrations of 20.0 and 30.0 mg/L, the biomass did not contain phycoerythrin. Copper oxide nanoparticles, although also inducing a reduction in the amount of phycobiliproteins in *Nostoc* biomass, caused a much more modest decrease. The lowest total phycobiliprotein content was recorded in biomass grown in the presence of 30.0 mg/L CuONPs, where phycobiliproteins constituted 10.06% of the biomass—25.2% lower than in the control. Notably, phycoerythrin was present in all experimental variants with CuONPs.

### 3.4 Modification of proteins, carbohydrates, lipids and malondialdehyde content in *Nostoc linckia* biomass under the influence of Cu and CuO nanoparticles

**Figure 4:**
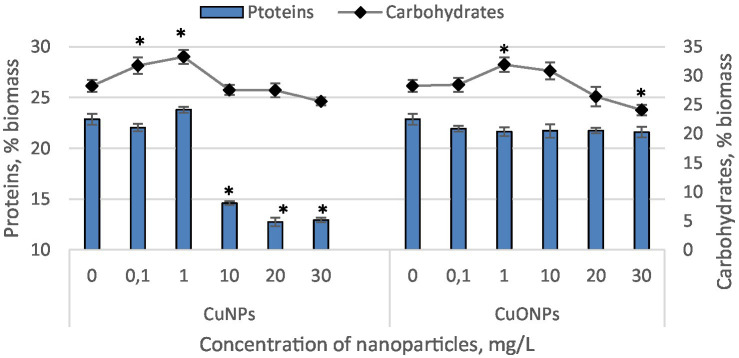
The effect of CuNPs and CuONPs on the quantity of proteins and carbohydrates in *Nostoc linckia* biomass (in % of dry biomass). *p < 0.05.

The quantities of proteins and carbohydrates in the biomass of *Nostoc linckia* during cultivation in the presence of different concentrations of copper and copper oxide nanoparticles are presented in Figure 4.

Copper nanoparticles at concentrations of 0.1 and 1.0 mg/L, as well as all tested concentrations of copper oxide nanoparticles, did not induce significant changes in the protein content of *Nostoc* biomass. The biomass from these experimental variants contained between 21.6 and 23.8% protein, values very close to those of the control, which contained 22.9% protein relative to dry biomass. In contrast, Nostoc biomass from variants with CuNPs concentrations of 10.0, 20.0, and 30.0 mg/L exhibited a significantly reduced protein level. At a concentration of 10.0 mg/L CuNPs, proteins constituted 14.6% of the dry biomass, which is 24.8% lower than the control. At concentrations of 20.0 and 30.0 mg/L, proteins accounted for 12.7 and 12.9% of the dry biomass, representing reductions of 44.3 and 43.5%, respectively, compared to the control. The carbohydrate content in *Nostoc* biomass varied relative to the control only in a few experimental variants. At CuNPs concentrations of 0.1 and 1.0 mg/L, carbohydrate content increased by 12.3 and 17.7%, respectively, compared to the control. In both cases, the differences were statistically significant (*p* = 0.045 and *p* = 0.002, respectively). Similarly, a 12.9% increase (*p* = 0.008) in carbohydrate content compared to the control was observed at a concentration of 1.0 mg/L CuONPs. However, at a concentration of 30.0 mg/L CuONPs, a decrease of approximately 14.8% (*p* = 0.022) in carbohydrate content was recorded. In all other experimental variants, the carbohydrate content in *Nostoc* biomass remained at the control level.

**Figure 5:**
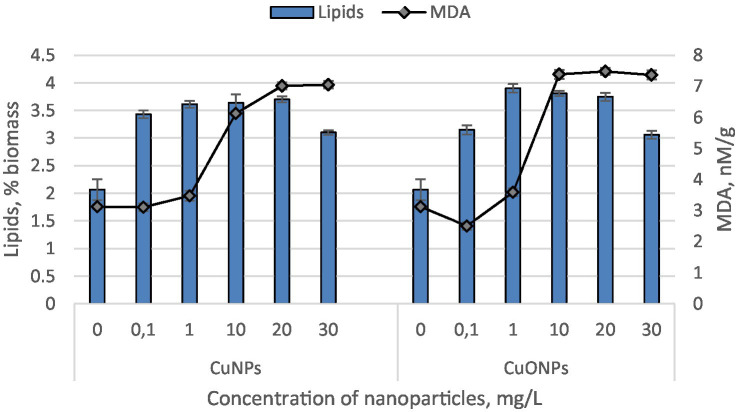
The effect of CuNPs and CuONPs on the quantity of lipids (in % of dry biomass) and malondialdehyde (MDA, in nM/G of dry biomass) in *Nostoc linckia* biomass. Except for the MDA value at a concentration of 0.1 mg/L CuNPs, all other values in the diagram show statistically significant differences compared to the control (*p* < 0.05).

The quantities of lipids and malondialdehyde (MDA) in the biomass of *Nostoc linckia* cultivated in the presence of various concentrations of copper and copper oxide nanoparticles are presented in Figure 5.

Both copper and copper oxide nanoparticles at the tested concentrations induced a significant increase in lipid content in the *Nostoc* biomass. Copper nanoparticles caused a lipid increase ranging from 50.1 to 79.2% compared to the control. Notably, there were no statistically significant differences among the experimental variants with CuNPs concentrations of 0.1, 1.0, 10.0, and 20.0 mg/L. Only the concentration of 30.0 mg/L resulted in lipid level changes distinct from the other variants, showing the smallest increase in the series—50.1% compared to the control. The level of MDA, a marker of oxidative stress and a product of lipid peroxidation, significantly exceeded the control values at CuNPs concentrations of 1.0, 10.0, 20.0, and 30.0 mg/L. At a concentration of 1.0 mg/L CuNPs, the MDA level increased by 11.1% compared to the control, while at the higher concentrations, it rose by 1.96, 2.25, and 2.26 times, respectively, relative to the control. Copper oxide nanoparticles, at all tested concentrations, increased lipid levels in the biomass by 48.1–88.9% compared to the control. The maximum lipid content was observed in experimental variants with CuONPs concentrations of 1.0, 10.0, and 20.0 mg/L. Under the influence of copper oxide nanoparticles, the MDA levels in *Nostoc* biomass were also affected. At a concentration of 0.1 mg/L CuONPs, the MDA content decreased by 19.9% compared to the control. However, in all other variants, the MDA level increased. At a concentration of 1.0 mg/L CuONPs, the increase was modest—14.9%, while at concentrations of 10.0, 20.0, and 30.0 mg/L, the MDA levels rose by 2.37, 2.40, and 2.36 times, respectively, compared to the control.

### 3.5 Morphological modification in *Nostoc linckia* induced by CuNPs and CuONPs

**Figure 6:**
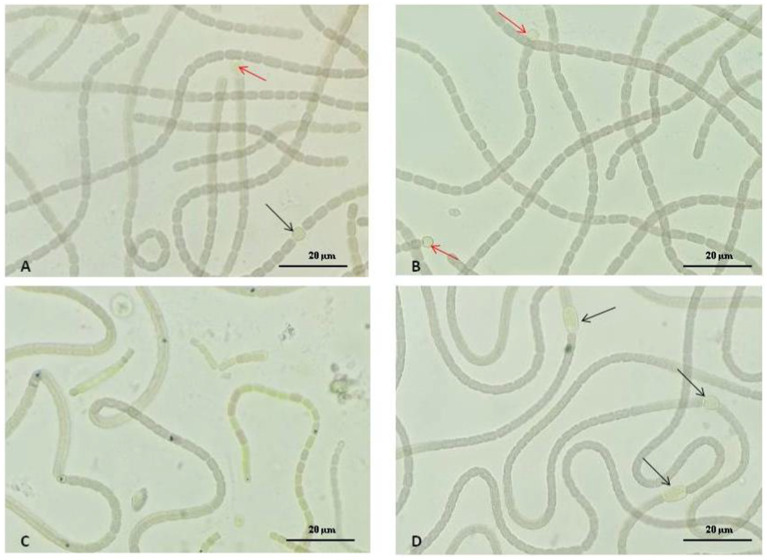
The effect of CuNPs and CuONPs on the morphology of *Nostoc linckia*. **(A,B)** Control samples; **(C)** Biomass grown under 30 mg/L of CuNPs; **(D)** Biomass grown under 30 mg/L of CuONPs. Black arrows indicate intercalary heterocysts, and red arrows indicate terminal heterocysts.

In Figure 6, images illustrate the control culture of *Nostoc linckia* and cultures grown with the addition of 30 mg/L nanoparticles of both types. In images (A and B), representing the control, long trichomes are clearly visible, composed of well-defined cells of approximately equal size with uniform coloration. Intercalary and terminal heterocysts of normal size and shape for this species are distinctly observed. In image (C), representing the Nostoc culture treated with copper nanoparticles at a concentration of 30 mg/L, fragmented trichomes are evident, with cells showing uneven and atypical coloration for the species. In image (D), larger heterocysts can be observed.

## 4 Discussion

The range of nanoparticle concentrations in this study was between 0.1 and 30 mg/L, selected based on the conventional classification of nanoparticle concentrations as ‘low’ or ‘high,’ depending on their mode of action on microalgae and cyanobacteria (Vargas-Estrada et al., 2020). At lower concentrations, nanoparticles can stimulate growth and biosynthesis through hormetic mechanisms (Rudi et al., 2025). In contrast, at higher concentrations, the stimulation of specific compound synthesis may result from oxidative stress induced in the cells (Wang et al., 2021). This range, therefore, encompasses both concentration domains, enabling a comprehensive evaluation of their potential effects on the cyanobacterium *Nostoc linckia*.

The contrasting effects of copper nanoparticles (CuNPs) and copper oxide nanoparticles (CuONPs) on biomass accumulation in *Nostoc linckia* reveal important insights into their interactions with cyanobacteria. The results demonstrate that at low concentrations of 0.1 and 1.0 mg/L, neither CuNPs nor CuONPs significantly impacted biomass accumulation compared to the control. However, starting from 10.0 mg/L, the two nanoparticle types exhibited distinct effects. While CuNPs caused a statistically significant reduction in biomass, CuONPs at the same concentration induced a notable biomass increase. A similar pattern was observed at 20.0 mg/L, with CuNPs causing a 23.0% biomass reduction and CuONPs leading to a 17.0% increase. At the highest concentration of 30.0 mg/L, CuNPs strongly inhibited biomass accumulation (24.6% reduction), whereas the stimulating effect of CuONPs disappeared, and the biomass level remained comparable to the control.

The divergent outcomes can be attributed to differences in the physicochemical properties and behavior of the two nanoparticle types. CuNPs, with their higher reactivity and faster release of copper ions, are likely to generate greater oxidative stress in *Nostoc linckia*, leading to disrupted cellular processes and inhibited growth. In contrast, CuONPs release copper ions more gradually, providing essential micronutrients at moderate concentrations (10–20 mg/L) and promoting metabolic activity and biomass accumulation. This stimulation effect suggests that CuONPs may be less toxic than CuNPs under similar conditions, though their benefits diminish at higher concentrations, possibly due to the accumulation of reactive oxygen species (ROS) or other stress-related effects that exceed the cyanobacterium’s adaptive capacity.

Literature data on the tolerance levels of microalgae to the action of Cu and CuO nanoparticles vary widely and are often contradictory, reflecting differences in experimental conditions and species-specific responses*. Nostoc linckia* appears to be significantly more tolerant to the effects of copper oxide nanoparticles compared to other phycological species. For instance, in cultures of the microalgae *Dunaliella salina, Thalassiosira weissflogii*, and *Prorocentrum cordatum*, inhibitory effects are observed at nanoparticle concentrations as low as 0.1 mg/L. According to the authors, this effect is attributed to the excessive production of reactive oxygen species (Solomonova E. S. et al., 2023; Solomonova E. et al., 2023). For *Skeletonema costatum* and *Nitzschia closterium*, the EC50 values ranged from 0.663 to 2.455 mg/L for nano-Cu (Huang et al., 2022). On the other hand, *Chlorella vulgaris* exhibited unchanged growth, slight production of reactive oxygen species, and significant membrane damage upon exposure to CuO nanoparticles in a concentration of 10 mg/L (Wang et al., 2020). This highlights the necessity of conducting targeted tests for each individual microalgal species to accurately assess their sensitivity and adaptation mechanisms.

The results highlight the remarkable efficiency of *Nostoc linckia* in accumulating copper from culture media when exposed to copper nanoparticles (CuNPs) and copper oxide nanoparticles (CuONPs). Notably, at lower concentrations (0.1, 1.0, and 10.0 mg/L), CuNPs were entirely absorbed by the biomass, as were CuONPs at 0.1 mg/L. Even at higher concentrations, both types of nanoparticles exhibited minimal residual copper in the culture liquid, reflecting the sustained bioaccumulation potential of *Nostoc linckia*. The cyanobacterium’s high removal rates suggest a robust adaptation to the presence of copper-based nanoparticles, likely involving mechanisms such as metal chelation by extracellular polymeric substances (EPS) and intracellular sequestration. The slightly lower removal efficiency at elevated nanoparticle concentrations (90.5–97.9%) could indicate partial saturation of the uptake mechanisms or stress-induced alterations in cellular processes. Comparing the two nanoparticle types, CuNPs and CuONPs appear to be similarly effective in terms of copper removal, particularly at lower concentrations where complete uptake was observed. Due to their oxidized nature and higher solubility, CuONPs release copper ions more rapidly than CuNPs, which must first undergo oxidation before ion release into the solution. However, the similar accumulation of copper by Nostoc in media containing CuONPs and CuNPs suggests the presence of compensatory mechanisms. One possible explanation lies in the differences in uptake and bioaccumulation processes. Smaller copper nanoparticles (25 nm) may be more easily internalized by cells through endocytosis or passive transport, facilitating intracellular copper accumulation (Wang et al., 2019). In contrast, CuONPs, being larger (50 nm), may remain predominantly extracellular, but their faster copper ion release could maintain a balanced uptake similar to that observed with smaller nanoparticles.

The data reveal that the pigment content of *Nostoc linckia* biomass responds distinctly to copper nanoparticles (CuNPs) and copper oxide nanoparticles (CuONPs). Under the influence of CuNPs, carotenoid content increased at lower concentrations (0.1–10.0 mg/L), peaking at 1.0 mg/L with a 15.1% rise. However, at higher concentrations (20.0 and 30.0 mg/L), carotenoid levels dropped significantly, indicating stress-induced degradation. A similar trend was observed for chlorophyll α, though the increase was statistically significant only at 10.0 mg/L, suggesting a threshold for beneficial stimulation. In contrast, CuONPs promoted a consistent increase in both pigments, with the highest enhancement at 20.0 mg/L (16.9% for chlorophyll α and 11.6% for carotenoids). This suggests that CuONPs at moderate concentrations may support photosynthetic processes by providing a less disruptive copper source compared to CuNPs.

Copper oxide nanoparticles (CuO NPs) significantly disrupt the photosynthetic apparatus in microalgae by inhibiting the oxygen-evolving complex (OEC) in the photosynthetic electron transport chain, leading to photoinhibition of PSII, reduced light energy utilization efficiency, and a decrease in photosynthetic pigment content. This disruption, caused by ROS accumulation and structural damage to chloroplasts, results in carbon starvation and growth inhibition in microalgae (Che et al., 2018). In *Ankistrodesmus densus*, Cu nanoparticles (Cu-NPs) at concentrations ranging from 0.3 to 635 μg/L caused a reduction in photosynthetic pigments. This effect is attributed to the dissolution of Cu-NPs in the medium, releasing Cu^2+^ ions, which were measured and used as a proxy for nanoparticle concentration (Barreto et al., 2021).

The phycobiliproteins (phycocyanin, allophycocyanin, and phycoerythrin) in *Nostoc linckia* biomass were significantly more sensitive to CuNPs than to CuONPs. CuNPs at 10.0–30.0 mg/L drastically reduced phycobiliprotein content, with concentrations dropping to less than 20% of the control levels. Notably, at 20.0 and 30.0 mg/L, phycoerythrin was entirely absent, indicating severe disruption to pigment biosynthesis or degradation pathways. In contrast, CuONPs caused a less pronounced decrease, with total phycobiliproteins reduced by only 25.2% at the highest concentration tested (30.0 mg/L), and phycoerythrin remained detectable in all samples. The strong inhibitory effects of CuNPs on phycobiliproteins and photosynthetic pigments may be harnessed to control harmful algal blooms.

The analysis reveals that the presence of copper nanoparticles (CuNPs) and copper oxide nanoparticles (CuONPs) has distinct impacts on the protein and carbohydrate content in the biomass of *Nostoc linckia*. For protein content, CuNPs at higher concentrations (10.0–30.0 mg/L) had a pronounced inhibitory effect. Significant reductions in protein levels—up to 44.3%—were observed, suggesting that CuNPs disrupt protein synthesis or accelerate protein degradation under stress. In contrast, lower concentrations of CuNPs (0.1 and 1.0 mg/L) and all tested concentrations of CuONPs did not affect protein levels, with values closely matching the control. Carbohydrate content was less consistently affected than protein levels. Low concentrations of CuNPs (0.1 and 1.0 mg/L) and CuONPs (1.0 mg/L) stimulated carbohydrate accumulation, with increases of up to 17.7% compared to the control. This could indicate a stress adaptation mechanism, where the cyanobacterium redirects metabolic resources toward carbohydrate production as a protective or storage strategy. However, at higher concentrations (30.0 mg/L), CuONPs led to a significant 14.8% reduction in carbohydrate content, indicating that stress levels exceeded the adaptive capacity of the cells. For all other concentrations, carbohydrate levels remained unchanged, reflecting minimal metabolic disruption. CuNPs exhibit a stronger inhibitory effect on protein content at higher concentrations, likely due to their greater tendency to induce oxidative stress. In contrast, CuONPs have a less disruptive influence, maintaining protein levels even at higher concentrations, but can negatively affect carbohydrate synthesis at extreme concentrations. The initial increase in carbohydrates at low nanoparticle concentrations suggests a potential metabolic adaptation to stress, where cells prioritize energy storage or protective polysaccharides. The ability of CuONPs to maintain stable protein levels and stimulate carbohydrate production at moderate concentrations could be exploited to enhance the nutritional quality of Nostoc-derived biomass for use in aquaculture and biofertilizers.

Both CuNPs and CuONPs induced significant lipid accumulation in *Nostoc linckia*, suggesting that lipid biosynthesis is a prominent stress-response pathway. CuNPs increased lipid content by 50.1 to 79.2%, with the most pronounced increases observed at concentrations of 0.1–20.0 mg/L. Interestingly, there were no significant differences in lipid levels across these concentrations, indicating that moderate CuNPs doses similarly stimulate lipid production. At the highest concentration (30.0 mg/L), the lipid increase was the smallest (50.1%), possibly reflecting a threshold beyond which metabolic pathways are disrupted. For CuONPs, lipid levels increased by 48.1 to 88.9%, with the highest values recorded at concentrations of 1.0, 10.0, and 20.0 mg/L.

The modification of the content of biomolecules in the biomass of microalgae and cyanobacteria under the influence of copper and copper oxide nanoparticles is invoked in multiple publications (Barreto et al., 2019; Sibi et al., 2017; Barreto et al., 2021; Cavalletti et al., 2022; Che et al., 2018; Fazelian et al., 2020; Huang et al., 2022; Lau et al., 2022; Melegari et al., 2013; Nguyen et al., 2020; Vargas-Estrada et al., 2020; Wang et al., 2020), but in the absence of a common experimental plan and in the conditions of applying different types of nanoparticles, synthesized physico-chemically and biologically, of different sizes and stabilized in different ways, the results obtained differ from one experiment to another. The common elements consist of protein degradation at high concentrations and the accumulation of carbohydrates and lipids as a protective mechanism of microalgae and cyanobacteria under stress conditions.

MDA levels, a marker of oxidative stress and lipid peroxidation, provide insights into the oxidative damage caused by nanoparticle exposure. CuNPs induced significant increases in MDA content at concentrations of 1.0–30.0 mg/L, with the highest increases (up to 2.26 times the control) at 10.0–30.0 mg/L. These results indicate that CuNPs strongly promote oxidative stress at moderate to high concentrations, likely due to their generation of reactive oxygen species (ROS) or interaction with lipid membranes. CuONPs had a more nuanced effect on MDA levels. At 0.1 mg/L, MDA content decreased by 19.9%, suggesting a protective effect or enhanced antioxidant activity at this low concentration. However, at higher concentrations (10.0–30.0 mg/L), MDA levels increased significantly, peaking at 2.40 times the control at 20.0 mg/L. This reflects a threshold beyond which the antioxidant defenses of *Nostoc linckia* are overwhelmed, leading to significant lipid peroxidation. Both types of nanoparticles induced oxidative stress at higher concentrations, as evidenced by increased MDA levels. CuNPs caused oxidative stress at lower concentrations (1.0 mg/L), whereas CuONPs showed protective effects at 0.1 mg/L, suggesting a more gradual onset of stress with CuONPs. The ability of CuONPs to stimulate lipid accumulation at moderate concentrations could be exploited in biotechnological applications such as biofuel production, where enhanced lipid yields are desirable. The oxidative stress induced by nanoparticles, as measured by MDA levels, provides insights into safe dose thresholds for environmental or industrial applications involving cyanobacteria. Understanding these dose-dependent effects is essential for optimizing nanoparticle use while minimizing adverse impacts on cyanobacterial systems and ecosystems.

Oxidative stress in the case of contact of CuNPs and CuONPs with microalgae and cyanobacteria, according to many researchers, is caused by the release of ions, which occurs much faster in the case of copper nanoparticles. It is known that redox reactions with the participation of copper ions are the main generators of reactive oxygen species in living cells. However, there are data demonstrating that, in certain circumstances, the cytotoxic effects attributed to the released copper fraction are significantly less pronounced than those caused by the nanoparticles themselves (Midander et al., 2009). Thus, in the case of the interaction of living cells with copper nanoparticles and copper oxide nanoparticles, we should take into account the direct interactions of nanoparticles with membranes and other cellular structures, including the shadowing effect of NPs.

The analysis of the morphological changes in *Nostoc linckia* cultures treated with copper nanoparticles (CuNPs) and copper oxide nanoparticles (CuONPs) reveals several important insights regarding the impact of these nanoparticles on cyanobacterial structure and growth patterns. In the control culture, *Nostoc linckia* maintains its typical morphology, characterized by long trichomes formed by well-defined cells of uniform size. The cells have consistent coloration, indicative of normal physiological conditions. The presence of intercalary and terminal heterocysts is clearly visible, with these specialized cells showing typical size and shape. This serves as the baseline for comparison, illustrating the healthy and stable state of *Nostoc linckia* in the absence of external stressors. In cultures treated with 30 mg/L of CuNPs (Image C), noticeable changes in morphology are evident. The trichomes are fragmented, which could indicate cellular damage or disruption of trichome cohesion. The cells within the trichomes display uneven and atypical coloration compared to the control, suggesting that CuNPs are causing physiological stress, possibly related to oxidative damage or impaired pigment synthesis. In the culture treated with CuONPs at the same concentration (30 mg/L, Image D), there are some notable differences compared to the CuNP-treated culture. While trichomes still appear fragmented, there is a more pronounced presence of larger heterocysts which are typically involved in nitrogen fixation and survival under adverse environmental conditions.

The results obtained from the exposure of *Nostoc linckia* to copper nanoparticles (CuNPs) and copper oxide nanoparticles (CuONPs) provide an understanding of how these nanoparticles interact with biological systems at the cellular structure and function level. The observed changes in biomass accumulation, pigment content, proteins, carbohydrates, lipids, and malondialdehyde (MDA), as well as morphological modifications, suggest a series of physiological and biochemical responses to the stress induced by nanoparticles. Copper, in its nanoparticulate forms, induces significant oxidative stress, leading to increased MDA levels, modifications in pigment production and structural substances like proteins and carbohydrates. On the other hand, copper oxide nanoparticles appear to stimulate biomass growth and alter pigment structure differently compared to CuNPs, suggesting possible complementary or adaptive effects. This indicates that CuONPs have less toxicity at higher concentrations, and *Nostoc linckia* may exhibit adaptive mechanisms that allow survival and even growth under exposure to these particles.

Practically, these results are essential for the application of copper nanoparticles in fields such as bioremediation, agriculture, and biotechnology*. Nostoc linckia’*s ability to accumulate copper from the environment suggests that this species could be used for cleaning waters contaminated with heavy metals through copper absorption and fixation. Additionally, the morphological and biochemical changes induced by copper nanoparticles can be exploited to optimize biomass and pigment production processes, which are critical in the production of biofertilizers or high-value additives, such as natural colorants. On the other hand, these results underline the importance of regulating the concentrations of copper nanoparticles used in natural or industrial environments, considering that prolonged exposure to high concentrations of nanoparticles can lead to negative effects on various organisms, including inhibition of biomass growth and cellular structural damage. Therefore, it is crucial to balance the potential benefits of copper nanoparticles with ecological and health risks in their industrial and ecological applications.

## 5 Conclusion

This study investigates the effects of CuNPs and CuONPs on *Nostoc linckia*, focusing on critical biological parameters such as biomass accumulation, biochemical composition, pigment production, and cellular structural integrity. By exposing *Nostoc linckia* to varying concentrations of these nanoparticles, ranging from 0.1 to 30 mg/L, this research evaluates the differential responses induced by CuNPs and CuONPs. Parameters such as protein, lipid, and pigment content, along with markers of oxidative stress like malondialdehyde (MDA), were analyzed to understand the biochemical alterations. In addition, copper bioaccumulation was assessed to quantify the uptake and retention of this metal in the cyanobacterial cells. Microscopic analyses were also performed to detect structural modifications, including changes in trichome integrity and chromaticity in the case of copper nanoparticles, and heterocyst characteristics in case of copper oxide nanoparticles.

Preliminary observations reveal contrasting effects of the two nanoparticle types. While CuNPs exposure led to reductions in biomass, protein levels, and pigments, it significantly increased lipid and MDA content, indicating oxidative stress and metabolic disruption. Similarly, CuONPs caused a marked increase in lipid and MDA levels, suggesting oxidative stress despite the comparatively moderate alterations in other biochemical parameters. Both nanoparticle types, however, caused notable bioaccumulation of copper and structural modification in *Nostoc linckia* cells, highlighting the impact of nanoparticle composition on cellular responses.

These findings underscore the need to further explore nanoparticle-microbe interactions, as they offer valuable insights into the dual role of nanoparticles as stressors and stimulators in microbial systems. The distinct impacts of copper and copper oxide nanoparticles on cyanobacteria provide a foundation for optimizing their applications in biotechnology while addressing environmental considerations associated with nanoparticle use.

## Data Availability

The raw data supporting the conclusions of this article will be made available by the authors, without undue reservation.
